# Highly Zeolite-Loaded Polyvinyl Alcohol Composite Membranes for Alkaline Fuel-Cell Electrolytes

**DOI:** 10.3390/polym10010102

**Published:** 2018-01-22

**Authors:** Po-Ya Hsu, Ting-Yu Hu, Selvaraj Rajesh Kumar, Chia-Hao Chang, Kevin C.-W. Wu, Kuo-Lun Tung, Shingjiang Jessie Lue

**Affiliations:** 1 Department of Chemical and Materials Engineering, and Green Technology Research Center, Chang Gung University, Guishan District, Taoyuan City 333, Taiwan; mini50636@gmail.com (P.-Y.H.); hu70308@gmail.com (T.-Y.H.); rajeshkumarnst@gmail.com (S.R.K.); 2 Department of Chemical Engineering, National Taiwan University, Da-an, Taipei City 106, Taiwan; r04524057@ntu.edu.tw (C.-H.C.); kevinwu@ntu.edu.tw (K.C.-W.W.); kuolun@ntu.edu.tw (K.-L.T.); 3 Department of Radiation Oncology, Chang Gung Memorial Hospital, Guishan District, Taoyuan City 333, Taiwan; 4 Department of Safety, Health and Environmental Engineering, Ming-Chi University of Technology, Taishan District, New Taipei City 243, Taiwan

**Keywords:** zeolite composite, polymer electrolyte, ionic conductivity, direct alcohol fuel-cell performance

## Abstract

Having a secure and stable energy supply is a top priority for the global community. Fuel-cell technology is recognized as a promising electrical energy generation system for the twenty-first century. Polyvinyl alcohol/zeolitic imidazolate framework-8 (PVA/ZIF-8) composite membranes were successfully prepared in this work from direct ZIF-8 suspension solution (0–45.4 wt %) and PVA mixing to prevent filler aggregation for direct methanol alkaline fuel cells (DMAFCs). The ZIF-8 fillers were chosen for the appropriate cavity size as a screening aid to allow water and suppress methanol transport. Increased ionic conductivities and suppressed methanol permeabilities were achieved for the PVA/40.5% ZIF-8 composites, compared to other samples. A high power density of 173.2 mW cm^−2^ was achieved using a KOH-doped PVA/40.5% ZIF-8 membrane in a DMAFC at 60 °C with 1–2 mg cm^−2^ catalyst loads. As the filler content was raised beyond 45.4 wt %, adverse effects resulted and the DMAFC performance (144.9 mW cm^−2^) was not improved further. Therefore, the optimal ZIF-8 content was approximately 40.5 wt % in the polymeric matrix. The specific power output was higher (58 mW mg^−1^) than most membranes reported in the literature (3–18 mW mg^−1^).

## 1. Introduction

Fuel-cell technology is recognized as a promising electrical energy generation system for the twenty-first century, which supplies efficient and clean energy [1]. Natural and synthetic polymers have been used as efficient polymeric electrolytes for electrochemical applications due to their environmental friendliness, good thermal and mechanical strength, transparency upon film formation, and low cost [2,3,4,5]. Direct methanol fuel cells (DMFCs) are promising energy resources for portable devices, transportation and discrete power-generation systems. With the advantages of high volumetric energy density, low CO_2_ emissions and direct liquid fuel feed (with easy storage and delivery benefits), DMFCs are an attractive alternative to conventional energy systems.

The proton-exchange membrane (e.g., the Nafion membrane) is a well-known membrane material for fuel-cell applications. It has high mechanical properties and high proton conductivity [6]. However, there are several disadvantages including methanol crossover through the electrolyte membrane, which poisons the catalyst (Pt) due to CO species formation [7]. Many researchers used alkaline anion-exchange membranes (AEMs) for direct methanol alkaline fuel cells (DMAFCs) to overcome these obstacles [8,9]. In AEMs, the charge carriers are OH^−^ ions rather than H^+^ ions, thus they work under alkaline conditions where the electrochemical reactions are more facile than those in acidic medium [9,10,11]. The main advantages of DMAFCs include hydroxide ions moving crossflow to the methanol. This suppresses methanol diffusion through the membrane. Faster methanol oxidation rate is achieved in an alkaline medium compared to that in acidic solution [10,11]. The cell cost can be reduced since non-platinum catalysts (silver, nickel and palladium) can be used in alkaline mediums [12,13]. Consequently, alkaline fuel cells have received increasing attention over the last few years [9,14].

Several anion-exchange polymeric membranes [15,16] or hydroxide-conductive electrolytes (alkaline-doped neutral membranes) [12,17] offer cost advantages compared to the proton-exchange membranes. Polyvinyl alcohol (PVA) is an inexpensive polymer with an excellent film-forming ability [5] used in membrane separation [18], as a fuel-cell electrolyte [19] and in biological applications [20]. PVA has a repeating hydroxyl group that makes it hydrophilic and offers good compatibility in aqueous solutions. 

PVA induces extensive swelling in water, which decreases its mechanical stability [21]. One way to improve membrane stability and mechanical properties in aqueous solutions is to incorporate an inorganic filler into the polymer matrix [13,22]. In our previous work, we reported on the incorporation of fumed silica (FS) nanoparticles into a PVA matrix to improve the stability in aqueous solution. The physical nanoparticle crosslinking mechanism suppressed membrane dissolution in water [23]. In addition, FS particles inhibited polymer crystallization and enlarged the free volume size [24], which facilitated water molecule and hydroxide ion permeation, while hindering methanol transport [25]. In addition, PVA and PVA composites show good stability after doping with a potassium hydroxide (KOH) solution [26]. Fu et al. [27] reported that PVA–KOH membranes form hydrogen bonding between the OH and C–O groups on PVA and KOH molecules, which results in improved ionic conductivity and chemical stability. The ionic conductivities of the KOH-doped PVA/FS (0.058 S cm^−1^) increased significantly compared with pristine PVA (0.018 S cm^−1^), and the cell performance (39 mW cm^−2^ vs. 23.5 mW cm^−2^) also increased [26].

The inorganic fillers in a polymer matrix always present a load limit. Lue et al. [23] prepared mixed matrix membranes (MMMs) of PVA and commercial FS powders and found that the mechanical properties decreased at 30% FS loading in the PVA. The MMM became brittle and defects and/or cracks tended to occur at this high filler content. The optimal loading in that work was 20% FS content. Wu et al. [28] mentioned that the cell performance increased with level of carbon nanotubes (CNTs) loaded up to 0.15 wt %. With more CNTs in the polybenzimidazole (PBI) matrix, less free volume and a more tortuous path occurred for hydroxide to pass through the PBI matrix, resulting in a detrimental impact on cell performance. Ahn et al. [29] indicated the formation of non-porous particle (i.e., FS) aggregates in a polymer matrix resulting from the poor nanofiller distribution in the MMMs, which might lead to void volume between the aggregates and polymer. Therefore, making a homogenous polymer/inorganic membrane with higher filler loads is exceedingly important for membrane formation. Deng et al. [30] developed an effective approach to make well-dispersed PVA/zeolitic imidazolate framework-8 (ZIF-8) MMMs for ethanol and water pervaporation. In their study, the ZIF-8 was synthesized via an aqueous media and the ZIF suspension was compatible with PVA solution. The prepared MMMs contained up to 40 wt % ZIF-8 in the PVA membrane, with a dense film structure and homogeneous micromorphology without interfacial voids in the membrane. They reported that in ethanol aqueous solution pervaporation, the permeability and separation factor increased with the ZIF-8 content and the highest performance was achieved with 40 wt % ZIF-8 load. They indicated that the increased permeability and separation factors were attributed to the increased free volume for water to transport, and the molecular sieving nature of this porous ZIF-8 to prevent ethanol from permeation. The water-based ZIF-8 nanoparticles showed good compatibility with hydrophilic PVA polymer even at high filler concentrations.

**Figure 1:**
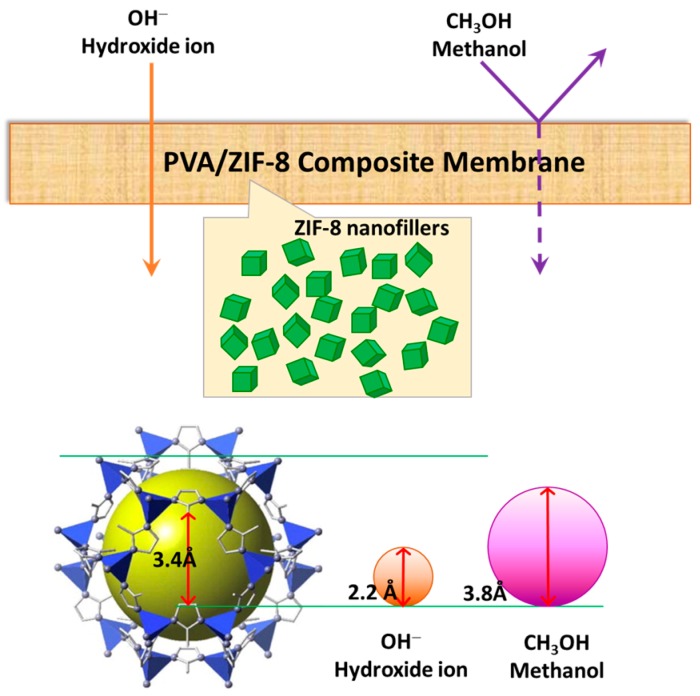
Illustration showing polyvinyl alcohol/zeolitic imidazolate framework-8 (PVA/ZIF-8) composite membrane with molecular screening effect: easy penetration of smaller hydroxide ions and suppression of larger methanol molecules. The methanol may transfer through the ZIF-8 external surface but has a limited diffusion rate in PVA matrix.

In this work we further investigate the efficiency of these PVA/ZIF-8 composites in DMAFC applications. As shown in Figure 1, ZIF-8 is a porous material in a subclass of metal–organic frameworks (MOFs) [31]. Its three-dimensional structures were constructed from tetrahedral metal ions (e.g., Zn, Co) bridged by imidazolate (Im) [32]. The Si–O–Si bonding angle in conventional zeolites and the metal–imidazole–metal in ZIFs are nearly the same. ZIFs have the advantages of zeolites, which exhibit high surface areas, high crystallinities, and thermal and chemical stabilities [33]. ZIFs also show good compatibility with polymers [34] owing to their imidazole groups. ZIFs have been widely applied to many fields, such as pervaporation [21,30], electronic devices [35], gas separation [36,37], catalysis [38] and hydrogen fuel cells [39,40]. ZIF-8, one of the most studied ZIFs, also demonstrates high thermal stability and remarkable chemical resistance [41]. The cavity size of ZIF-8 is 3.4 Å [42], which is between the kinetic diameter values of water (2.96 Å) and methanol (3.8 Å) [43] and can form a molecular sieve for these two components. Hydroxide ions have an effective radius of 1.10 Å [44] (diameter of 2.20 Å), slightly smaller than water molecules. It is therefore proposed that these MMMs can allow water and hydroxide ion diffusions but retard methanol transport (as shown in Figure 1), therefore having potential as an electrolyte material.

Libby et al. [45] synthesized PVA/mordenite composite membranes after doping with sulfuric acid for potential DMFC electrolytes. They reported that mordenite particles suppressed methanol passing through the membranes, which is similar to the molecular sieving effect. Meanwhile, the addition of zeolite decreases the proton conductivity of the membrane due to the proton transportation only taking place though the polymer phase, which was predicted using Maxwell’s theory. The selectivity (the ratio of proton conductivity to methanol permeability) of the PVA/mordenite membrane was significantly improved and the highest selectivity was at 50% (by volume) mordenite load in the composite membrane. However, no cell performance was conducted by these authors to confirm this loading effect.

In this study, we prepare PVA/ZIF-8 membranes with loads up to 45.4 wt % in order to achieve high ionic conductivity and suppressed methanol permeability. Compared to mordenite filler, the ZIF-8 mesoporous particles have smaller particle size (60 nm vs. 2–4 µm) and narrower cavity pore size (3.4 Å and >7 Å). The water-based ZIF-8 synthesis protocol without drying allows the nanofillers to disperse uniformly in the polymer matrix. Various amounts of ZIF-8 suspensions were added into the PVA solution to manipulate filler load and to investigate the filler content effect on membrane properties. The pristine PVA and PVA/ZIF-8 composite membranes were doped with 6 M KOH solution to form membrane electrolytes. DMAFC performance was measured and correlated to the membrane characteristics.

## 2. Materials and Methods 

### 2.1. ZIF-8 Synthesis and PVA/ZIF-8 Composite Preparation

The ZIF-8 nanoparticles were synthesized from 2-methylimidazole (≥98.0% purity, from Sigma-Aldrich, St. Louis, MO, USA) and zinc nitrate hexahydrate (≥99.0%, from J.T. Backer, Philipsburg, NJ, USA) in aqueous solution [30]. The PVA (molecular weight of 146–186 kDa, more than 99% hydrolyzed, Sigma-Aldrich) aqueous solution was added into the ZIF-8 suspension to form PVA/ZIF-8 slurries with various ZIF contents. The PVA/ZIF-8 mixture solution was poured onto a glass plate and cast with an application knife (at a clearance of 600 μm). The PVA/ZIF-8 composites were dried in a vacuum oven at 80 °C overnight.

### 2.2. Physical–Chemical Properties of ZIF-8 Particles and Membranes

The ZIF-8 powders, PVA and PVA/ZIF-8 membrane morphologies were analyzed using a field emission scanning electron microscope (FESEM, model JSM-7500F, Hitachi High-Technologies Corp., Tokyo, Japan) after the samples were freeze-fractured in liquid nitrogen and sputtered with gold. The membrane functional groups were characterized using a FTIR spectrometer (Model Spectrum 100, Perkin-Elmer Inc., Shelton, CT, USA) in the 4000 to 450 cm^−1^ range. The crystal characteristics of the PVA and PVA/ZIF-8 composite membranes were analyzed using X-ray diffraction (XRD, model D5005D, Siemens AG, Munich, Germany) with Cu Kα (wavelength of 1.54 Å) anode operating at 40 kV and 40 mA. The membrane was measured from angles of 5° to 30° at a scanning rate of 0.5° per second with a resolution of 0.02°. The degrees of PVA and PVA/ZIF-8 composite membrane polymer crystallinities were evaluated using a differential scanning calorimeter (DSC, Perkin-Elmer Inc., Shelton, CT, USA). The tested sample was heated from 25 °C to 300 °C at a scanning rate of 5 °C min^−1^ under a nitrogen atmosphere [23,46]. The degree of crystallinity *χ_C_* was calculated using the following equation:

(1) $$\chi_{C}=\frac{\Delta H}{\Delta H_{C}\left(1-\varphi_{z}\right)},$$

where *ϕ_z_* is the weight percent of ZIF-8 in the composite, Δ*H_C_* is the melting enthalpy of the completely crystallized PVA [47], and Δ*H* is the measured melting enthalpy of the composite.

Alkali uptake was used to determine the hydroxide absorbed on the membrane. The dry membrane was immersed in 6 M KOH solution at room temperature for 12 h and the weight change between the dry weight (*W_i_*, in g) and the total weight (*W_tt_*, in g) was measured. The alkali uptake (*M*) was calculated using the following expression [28]:

(2) $$M=\frac{W_{tt}-W_{i}}{W_{i}\left(1-\varphi \right)},$$

where *ϕ* is the weight percentage of PVA in the composite membranes.

### 2.3. Electrolyte Conductivity

The through-plane ionic conductivity was measured using the alternate circuit (AC) impedance method according to our previous work [28] and modified from the literature [48,49]. The PVA and PVA/ZIF-8 composite films were immersed in a 6 M KOH solution (Sigma-Aldrich) for 12 h. The thickness increase upon KOH doping was measured. The alkali-doped membrane was clamped between two stainless-steel electrodes with a working area of 1.33 cm^2^, and placed in a T-shaped glass holder. The apparatus was placed in a chamber at 30 °C or 60 °C at a relative humidity of 99%. A potentiostat (Autolab PGSTAT-30, Eco Chemie B.V., Utrecht, The Netherlands) was used to analyze the AC impedance of the KOH-doped membrane. The tested sample was measured at a scan range of 100 kHz–100 Hz and an excitation signal of 10 mV. The electrolyte bulk resistance *R*_E_ (Ω) was calculated from the Nyquist plot [26]. The conductivity (*σ*) was calculated according to the following equation:

(3) $$\sigma =\frac{L}{R_{E}A},$$

where *L* is the membrane thickness (cm) and *A* is the working area of the stainless-steel electrode (cm^2^).

### 2.4. Methanol Permeability Measurement

Methanol permeability of the KOH-doped PVA and PVA/ZIF-8 membranes was evaluated using a side-by-side diffusion cell consisting of two compartment glass reservoirs (source and receiving reservoirs). The sample membranes were clamped between these two reservoirs. A 2 M methanol (prepared from 99.9% solvent, Acros Organics, Geel, Belgium) aqueous solution and DI water was filled into the source reservoir and the receiving reservoir, respectively. The methanol transport concentration to the water was analyzed using density/specific gravity meter (model DA-130N, Kyoto Electronics Manufacturing Co. Ltd., Kyoto, Japan) by sampling a small amount of the solution from the receiving compartment at time intervals. The methanol permeability was calculated from the slope of a concentration–time plot, according to the following equation [50]:

(4) $$\mathrm{permeability}=\frac{\mathrm{slope}\times V\times L}{A\times C},$$

where *V* is reservoir capacity, *L* is membrane thickness, *A* is effective membrane area, and *C* is initial feed methanol concentration.

### 2.5. Cell Performance Measurement

Platinum–ruthenium on carbon spheres (HiSpec^TM^4000, 50 wt %, Pt:Ru = 1:1) and platinum on carbon spheres (40 wt %, Pt/C) catalysts were purchased from Johnson Matthey, Royston Hertfordshire, UK, and Tanaka, Tokyo, Japan, respectively. Catalyst inks were prepared by mixing the catalysts, Nafion binder solution (Sigma-Aldrich), isopropyl alcohol (Mallinckrodt Inc., Hazelwood, MO, USA), and DI water. These catalysts inks were sprayed onto carbon cloth (W0S1002, CeTech Co. Ltd., Taichung, Taiwan) resulting in catalyst load of 2 mg cm^−2^ of Pt–Ru for the anode and 1 mg cm^−2^ of Pt for the cathode [46]. The resulting gas diffusion electrodes were cut into 1.0 cm × 1.0 cm pieces. The KOH-doped PVA or PVA/ZIF-8 films (1.5 cm × 1.5 cm), with dry film thickness of 40 μm, were sandwiched between two electrodes to form a membrane electrode assembly (MEA) to evaluate the cell performance. To prevent liquid fuel from leaking, two Teflon gaskets with a hollow area of 1.0 cm × 1.0 cm were fixed between the MEA and flow field plates, which had carved flow channels facing the MEA. Copper-plated conductive end plates (thickness of 10 mm) were fixed next to the flow plates. The MEA, flow field plates and conductive end plates were firmly bolted and screwed using a torque wrench (torque of 392 N cm). The experimental fuel-cell testing setup was shown in our previous paper [12]. The 2 M methanol/6 M KOH solution as anode feed (flow rate of 5 mL min^−1^) was heated at 30 °C or 60 °C using a thermostatic chamber and recirculated through the anode compartment. The humidified oxygen gas as cathode feed (flow rate of 100 cm^3^ min^−1^) was fed directly into the cathode. An electrical load (PLZ164WA electrochemical system, Kikusui Electronics Corporation, Tokyo, Japan) was used to determine the current density (I) and potential (V) values at a scan rate of 0.01 V s^−1^. The power density (P) was the product of the current density (I) and cell voltage (V) values [46]. The peak power density (*P*_max_) under the tested condition was determined from the current density (P–I curve) plot recorded from the electrical load.

## 3. Results and Discussion

### 3.1. Morphology and Crystallinity of PVA and PVA/ZIF-8 Composites

**Figure 2:**
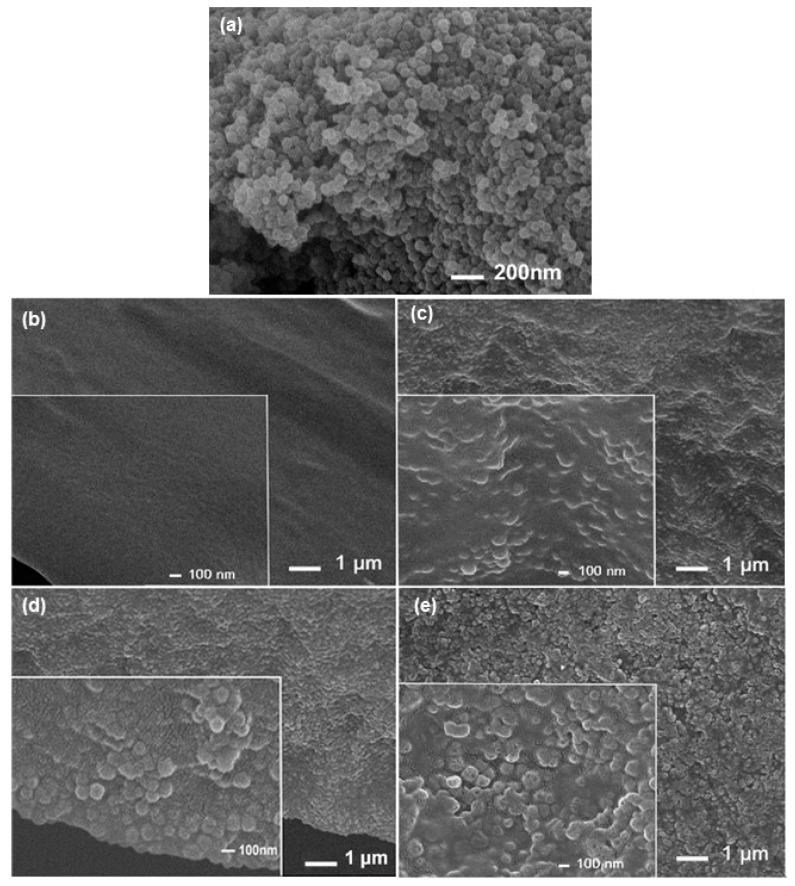
Field emission scanning electron microscope (FESEM) images of (**a**) ZIF-8 nanoparticles; and cross-sections of (**b**) pure PVA; (**c**) PVA/25.4% ZIF-8; (**d**) PVA/40.5% ZIF-8; and (**e**) PVA/45.4% ZIF-8 composites (insert figures show higher-magnification views of cross-sections of PVA/ZIF-8 composites).

The air-dried ZIF-8 nanoparticles ranged from 60 to 70 nm in diameter (Figure 2a) and tended to aggregate during the drying process. The as-synthesized nanoparticles were in aqueous solution and could form a compatible suspension and homogeneous composites with PVA, as illustrated in a recent publication [30]. The resulting PVA and PVA/ZIF-8 composites were dense films. The PVA surface was smooth and little difference was found on the surface morphology of PVA/ZIF-8 composites. The cross-sectional image of the as-prepared PVA membrane presents a smooth morphology (Figure 2b). From the cross-sectional views of PVA/ZIF-8 composite in Figure 2c–e, ZIF-8 particles are visible and the particle density obviously increased with the ZIF-8 content. In addition, no voids or cracks existed between the ZIF-8 nanoparticles and PVA polymer matrix. The ZIF water-based synthesis and its suspension mixed directly with PVA is beneficial for uniform ZIF-8 particle distribution in the PVA, preventing filler aggregation, usually found in the MMMs of PVA and air-dried ZIF particles [30] or FS nanoparticles at >20% load [12].

**Figure 3:**
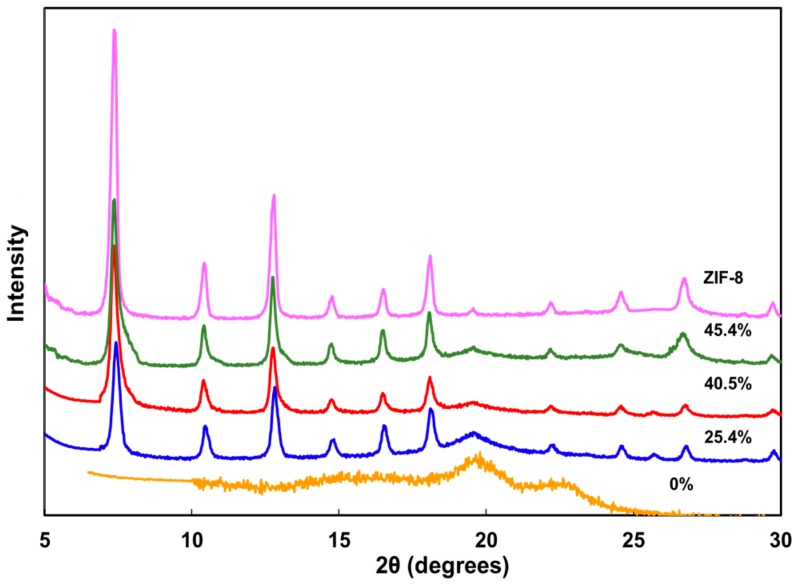
X-ray diffraction (XRD) patterns of PVA and PVA/ZIF-8 composites.

The XRD patterns of PVA/ZIF-8 composites are shown in Figure 3. Pure PVA has significant diffraction peaks at 2θ of 19.7° which are the main crystal peaks corresponding to a (101) reflection of the monoclinic crystal [51,52]. However, this peak intensity decreased with increasing ZIF-8 content. Many researchers found that incorporating inorganic particles into PVA resulted in lower XRD crystal diffraction intensity than the pristine PVA film [51,53,54]. Moreover, ZIF-8 nanoparticles revealed strong peaks at 7.3°, 10.3°, 12.7°, 16.4° and 18.0° [55] (Figure 3). As more ZIF-8 particles were mixed into the polymer matrix, these ZIF-characteristic peaks became dominant. The XRD patterns reflect the relative amount of ZIF-8 doped into the composites.

The PVA/ZIF-8 composite membranes were examined using DSC to characterize the crystal melting behavior. The PVA exhibited a significant endothermic peak at 215–225 °C. With increasing amounts of ZIF-8 particles in the composites, the polymer melting enthalpy of the composites decreased. The crystallinities were calculated after taking into account the ZIF-8 weight fractions. The polymer crystallinity decreased from 38.1% for the pristine PVA to 31.3% for PVA/45.4% ZIF-8 (Table 1). The ZIF-8 nanoparticles in the PVA may prevent the polymer chains from packing and aligning [26], resulting in less crystal segments and more amorphous regions.

### 3.2. Alkali Uptake and Ionic Conductivity of KOH-Doped PVA and PVA/ZIF-8 Composites

The alkaline uptakes of the as-prepared PVA and PVA/ZIF-8 composite membranes are shown in Table 1. The amount of KOH solution uptake increased approximately with increased addition of ZIF-8 particles up to 40.50% load, from 0.92 to 1.072 g g^−1^. This facilitated KOH uptake may be associated with the decreased polymer crystallinity and increased free volume in the amorphous regions. At a ZIF load of 45.40%, the PVA chain mobility is restricted and swelling confined, therefore the KOH uptake declined slightly to 0.998 g g^−1^.

The KOH-doped PVA and PVA/ZIF-8 composite membranes were measured for membrane resistance using an AC impedance analyzer at 30 °C and 60 °C. The data were converted into ionic conductivities, as summarized in Table 1. It is clear that all PVA/ZIF-8 composites present higher conductivities than the pure PVA, especially the PVA/40.5% ZIF-8 membrane with the highest value among the tested membranes. For the same composite, the ionic conductivity at 60 °C was higher than that at 30 °C. The PVA/40.5% ZIF-8 composite exhibited higher ionic conductivity than PVA/45.4% ZIF-8. This trend was the combined result from alkali uptake, the polymer crystallinity [23] and chain mobility [24,56]. When the PVA frameworks were mixed with ZIF-8, the continuous polymer phase provided hydroxyl groups for ionic diffusion. Such a framework interacts with polymer chains to interrupt polymer crystal formation and releases more amorphous regions for KOH swelling and ion transfer, enhancing ionic conductivity. However, as the ZIF load is increased beyond a certain threshold, the continuous polymer coverage could not be maintained and the membrane integrity was adversely affected. Based on the findings in this work and a previous study [30], the threshold was about 40 wt % by ZIF-8 weight, corresponding to 39.1% by volume.

**Figure 4:**
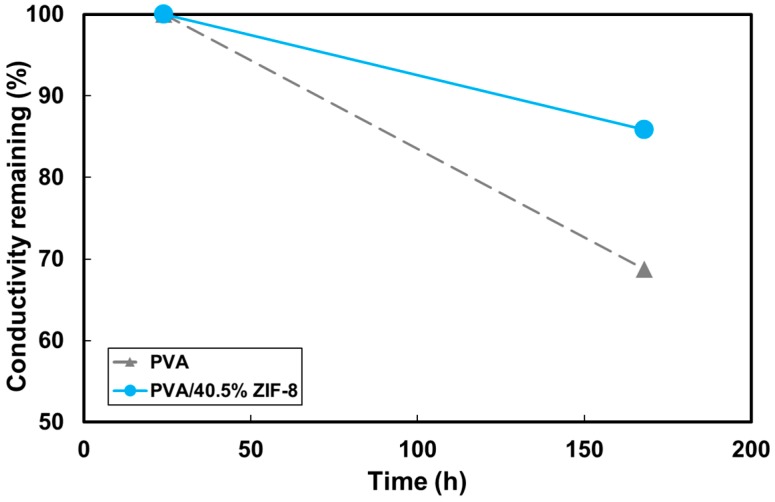
Alkaline stability presented in conductivity of PVA and PVA/40.5% ZIF-8 membranes as a function of time.

The alkaline stability of PVA and PVA/ZIF-8 composites was performed on the remaining conductivity after the PVA and PVA/40.5% ZIF-8 membranes were immersed into 6 M KOH for 24 and 168 h. Figure 4 shows the ionic conductivity of PVA and PVA/40.5% ZIF-8 membranes as a function of the immersion time. Both PVA and PVA/ZIF-8 membranes exhibited slightly decreased conductivity with time, probably due to less KOH retained on the membrane [57] due to dissolved polymer chains [23]. The conductivity of the pure PVA dropped 31.3% after 168 h. However, the PVA/ZIF-8 membrane conductivity showed less decline (14.1%) during the same lifespan. The ZIF nanoparticles could form a physical network structure in the PVA matrix, decreasing dissolution in water and maintaining polymer crystallinity [23]. The ZIF nanoparticles played an important role in preventing the polymer chains from unfolding by confined swelling [50]. The stable structure produced by the ZIF fillers may help KOH to remain around the polymer chains.

### 3.3. Methanol Permeability through KOH-Doped Membranes

**Figure 5:**
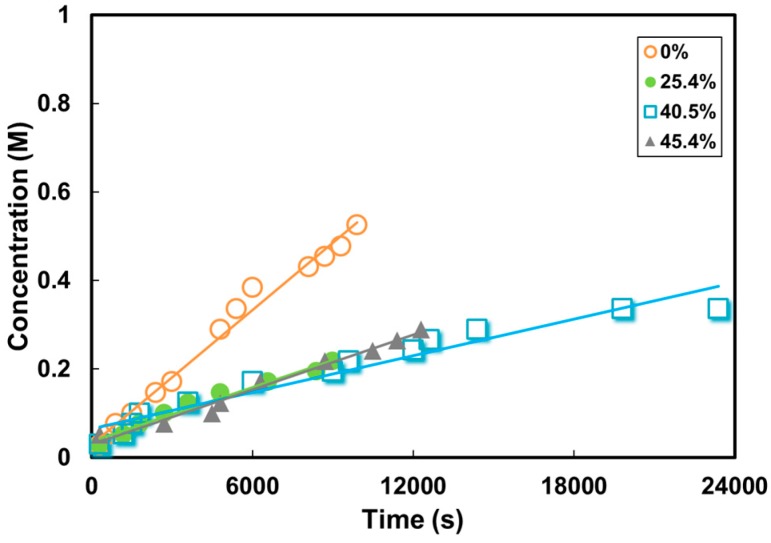
Change in methanol concentration in the receiving reservoir through PVA and PVA/ZIF-8 membranes as a function of time (volume of donor and receiving reservoir: 25 mL, membrane area: 0.785 cm^2^, temperature: 30 °C).

The time-resolved methanol concentrations permeated into the receiving reservoir during permeability testing are shown in Figure 5. The methanol permeability was calculated and shown in Table 1. The methanol permeability was 4.28 × 10^−6^ cm^2^ s^−1^ for the pure PVA, and decreased to 1.05 × 10^−6^ cm^2^ s^−1^ with ZIF-8 load of 40.50%. The obtained methanol permeability of the composite membranes is lower than that for the Nafion membrane (2.46 × 10^−6^ cm^2^ s^−1^) [58]. The decreased methanol permeability value indicates that the methanol solubility or the diffusion coefficient was reduced by the incorporation of ZIF-8 particles up to ZIF content of 40.50%. The kinetic diameter of methanol is about 0.38 nm [43]. The pore size of ZIF-8 was only 0.34 nm [42], which could allow water (kinetic diameter of 0.296 nm [43]) and hydroxide ion (effective ionic radius of 0.11 nm [44]) passage while limiting methanol molecule transport. Even methanol may transfer through ZIF-8’s external surface due to their similar solubility parameters (*δ* = 29.2–29.7 J^1/2^ cm^−3/2^ for methanol and *δ* = 29.5–31 J^1/2^ cm^−3/2^ for ZIF-8); the methanol has a limited diffusion rate in the PVA matrix [12]. As the ZIF content increased to 45.4%, the membrane permeability started to increase significantly. This may be related to the interfacial defects in the composite membrane. Such voids became leakage paths for methanol diffusion and the methanol permeability doubled in the PVA/45.4% ZIF-8 in comparison with the 40.5% composite.

Chung et al. [59] pointed out that MMMs could suffer from interfacial voids or a rigidified polymer layer at the interface between the soft polymer and rigid fillers, and increase small molecules’ permeability. These defects/cracks decrease membrane performance significantly. Bae et al. [60] and Mahdi et al. [61] demonstrated experimentally that interfacial voids formed at filler levels of 10 and 20 wt %, respectively, when polymers were mixed with dry fillers in the preparation of composites. Using the water-based method, we could obtain mixed matrix composites with a much higher filler load (>40 wt %) without sacrificing membrane integrity.

**Figure 6:**
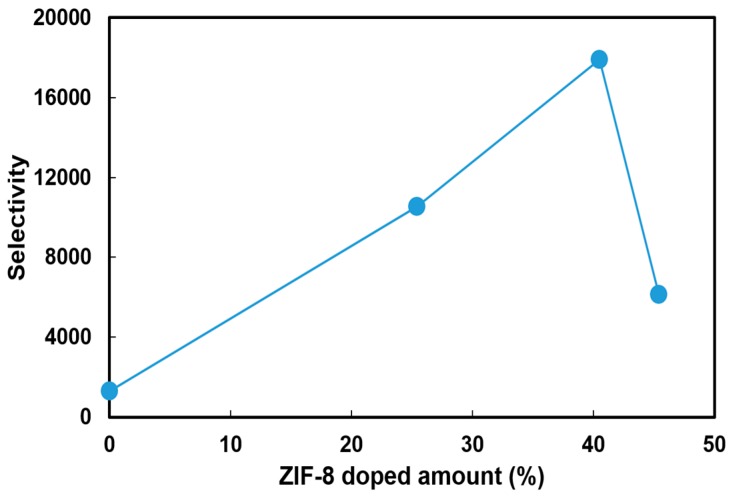
Selectivity (ratio of ionic conductivity to methanol permeability) of PVA and PVA/ZIF-8 membranes at 30 °C.

Figure 6 shows the selectivity value, defined as the ratio of ionic conductivity to the methanol permeability, at 30 °C for the PVA and PVA/ZIF-8 composite membranes. This parameter was used to predict the performance of electrolyte membranes in DMAFC. The incorporation of ZIF-8 particles was effective in increasing the selectivity. Moreover, PVA/40.5% ZIF-8 composites have the highest selectivity and we would expect that this composite would have the best performance in the fuel-cell power output.

### 3.4. Effect of ZIF-8 on Fuel-Cell Performance

**Figure 7:**
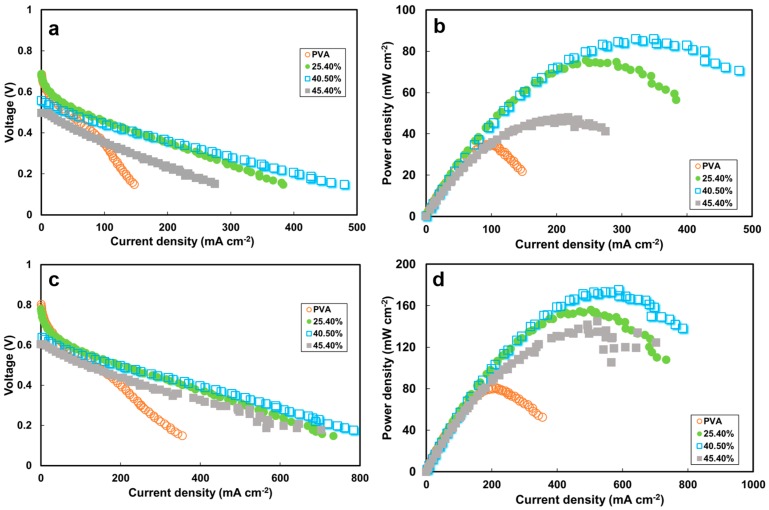
Direct methanol alkaline fuel cell (DMAFC) performance using various amounts of ZIF-8 in PVA electrolyte: (**a**) voltage; (**b**) power density as a function of current density at 30 °C; and (**c**) voltage; (**d**) power density at 60 °C. Gas diffusion electrodes: catalysts of 2 mg cm^−2^ Pt–Ru (1:1) for anode and 1 mg cm^−2^ Pt for cathode. Anode fuel: 2 M methanol + 6 M KOH at a flow rate of 5 mL min^−1^. Cathode fuel: humidified oxygen at a flow rate of 100 mL min^−1^.

The current density–cell voltage (I–V) and the power density–current density curves for a DMAFC with 2 M methanol/6 M KOH at 30 °C and 60 °C are shown in Figure 7. The open circuit voltage (*V*_oc_) values were 0.685, 0.687, 0.557 and 0.497 V for the PVA electrolyte membranes containing 0, 25.4, 40.5 and 45.4 wt % ZIF-8 (Figure 7a) at 30 °C, and the corresponding peak power density (*P*_max_) values were 34.8, 75.6, 85.9 and 48 mW cm^−2^ (Figure 7b), respectively. Obviously, increasing the ZIF-8 load was beneficial for obtaining high-power-density performance. The results at 60 °C cell temperature followed a similar trend. The *V*_oc_s were 0.801, 0.779, 0.637 and 0.604 V (Figure 7c), and the *P*_max_ values were 80.8, 155.7, 173.2 and 144.9 mW cm^−2^ with 0%, 25.4%, 40.5% and 45.4% ZIF-8 content, respectively (Figure 7d). The obtained cell voltage is higher than that for the Nafion 212 membrane (0.6 V) at the same operating temperature [58]. The peak power density increased with increasing operating temperature from 30 °C to 60 °C. Increasing the temperature can accelerate the electrochemical kinetics of the oxidation reaction at the anode and the reduction reaction at the cathode, generating more electrons within the same elapsed time [62]. The higher temperature also enhanced the ionic conductivity so the ohmic loss regions became lower, which slowed down the voltage loss and maintained the high power density. As the ZIF-8 load amount increased, the ohmic loss became lower at elevated ionic conductivity, and the membranes were resistant to methanol crossover (Figure 7a,c). These ZIF-8-containing electrolytes exhibited higher peak power density values (114%) than the pristine PVA (Figure 7b,d).

**Figure 8:**
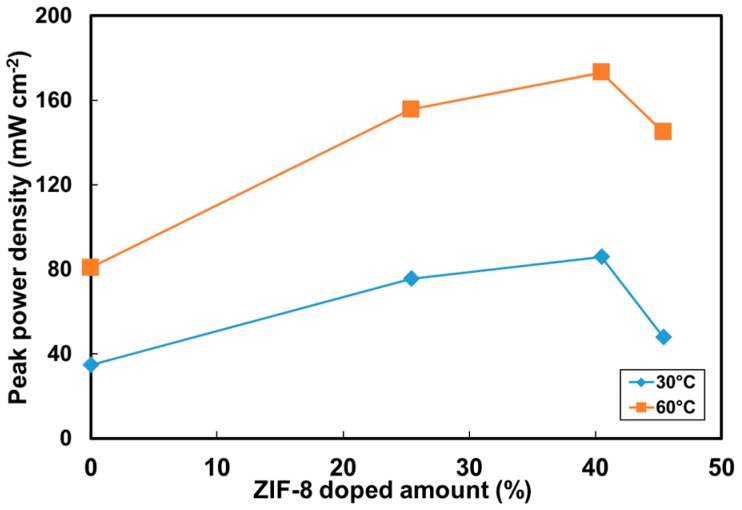
Effect of ZIF-8 loading on peak power density of DMAFCs. Gas diffusion electrodes: catalysts of 2 mg cm^−2^ Pt–Ru (1:1) for anode and 1 mg cm^−2^ Pt for cathode. Anode fuel: 2 M methanol + 6 M KOH at a flow rate of 5 mL min^−1^. Cathode fuel: humidified oxygen at a flow rate of 100 mL min^−1^.

The ZIF load effect on the peak power density is illustrated in Figure 8. The optimal performance was obtained with the membranes with a load of 40.5 wt % ZIF-8. As the load was increased to 45.4 wt % ZIF-8, the power density dropped. These ZIF-8-content effects on the fuel-cell performance can be explained in the aforementioned membrane characteristics. First of all, ZIF-8 incorporation into the PVA resulted in lower polymer crystallinities, which increased the amorphous phase and the free volume, allowing hydroxide ions to pass through PVA easily. Secondly, the easier hydroxide ions transfer through the PVA implies that higher ionic conductivity could be achieved. The high electrolyte conductivity directly affected the single-cell electrical resistance and the ohmic loss region of the I–V curve. As shown in the ohmic loss region in Figure 7c, the MEA resistance values were estimated to be 1.36 Ω for the cell consisting of the PVA and 0.50–0.58 Ω for those with the PVA/ZIF composite membranes. Thirdly, ZIF-containing membranes had lower permeability than the pristine PVA. Decreased methanol permeability value indicated that the methanol solubility and/or the diffusion coefficient was reduced. ZIF-8 addition causes a dilution effect on solvent uptake and the size-selectiveness [30] in the composite with increased polymer free-volume resulting in preferential transport of hydroxide ions while limiting methanol passing through the membranes. Therefore, the decreased fuel crossover can minimize the methanol oxidation potential on the cathode [63] and improve cell voltage. Fourthly, the optimal ZIF-8 load of 40.5 wt % can be predicted using the selectivity parameter as shown in Figure 6. Both selectivity (Figure 6) and power output (Figure 8) showed a consistent trend. As the load was increased above that threshold, adverse effects (such as decreased conductivity and higher methanol permeability) would result due to defects at the interfaces between the polymer and fillers. The fuel-cell power density then dropped significantly.

**Figure 9:**
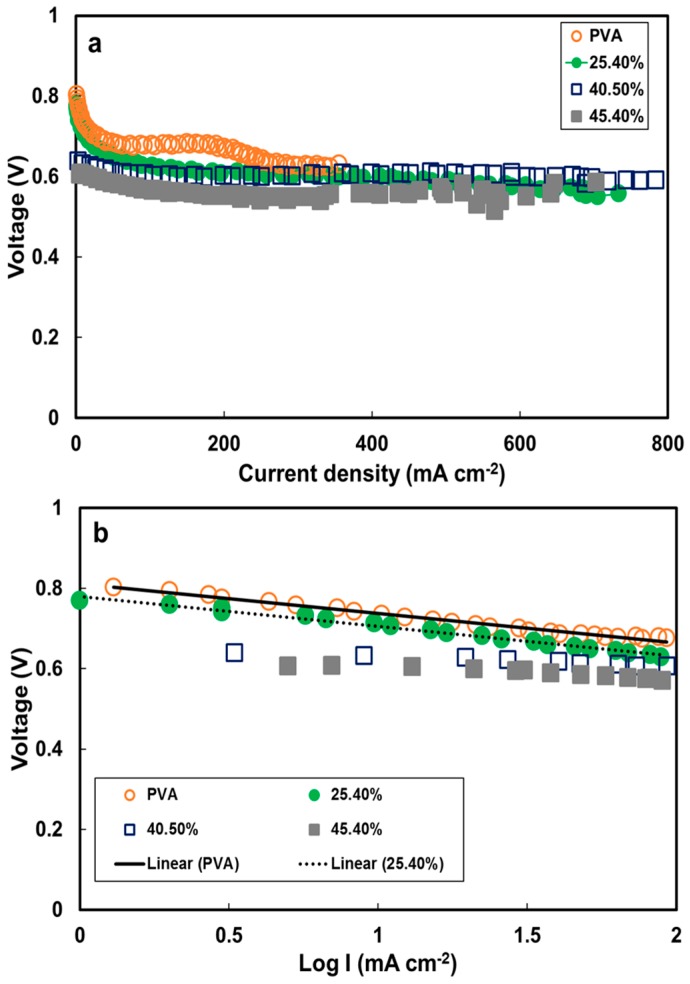
(**a**) IR-corrected polarization curves and (**b**) Tafel plots obtained from polarization curves (I is current density) of DMAFC performance using various amount of ZIF-8 in PVA electrolyte at 60 °C.

The IR-corrected polarization curves of the pristine PVA and PVA/ZIF-8 composite membranes are shown in Figure 9a. The trends of voltage loss associated with polarization curves are small as compared with internal resistance. Similar analysis was also reported by Xu et al. [64] for hydrogen fuel cells. The plots of IR corrected voltage vs. log I, which represent the tafel slope, for pristine PVA and PVA/ZIF-8 composite membranes are shown in Figure 9b. The Tafel slopes of the PVA composites were approximately 74–80 mV dec^−1^. These values are close to the Tafel slope values for the methanol oxidation on Pt–Ru (96 mV dec^−1^ [65]) and oxygen reduction reaction on Pt (60–120 mV dec^−1^ [66]). Li et al. reported that as long as the methanol permeability lies below a certain threshold, its impact on power output diminished. On the contrary, membrane ionic conductivity plays a major role in resulting power density [67]. The PVA/40.50% ZIF-8 electrolyte conductivity (shown in Table 1) contributes to the high power density in this work.

Table 2 shows a summary of the *P*_max_ values for DMAFCs using various membranes as reported by our group and in other literature. The *P*_max_ values ranged from 6 to 174 mW cm^−2^ [13,25,26,27,50,68,69] and from 35 to 200 mW cm^−2^ using PVA- and quaternized polyvinyl alcohol (QPVA)-based electrolytes, respectively [46,62,70,71,72]. Although QPVA-based membranes contained ammonium functional groups to facilitate ion conduction [46], the high *P*_max_ values obtained using ZIF-8 nanofillers in this work approach those of QPVA membranes, even with reduced catalyst load (Table 2). The specific power output (i.e., generated *P*_max_ normalized to catalyst load) is higher than most literature data. This water-based filler synthesis proved to be an effective route for preparing composites containing high nanoparticle load in hydrophilic polymers.

**Figure 10:**
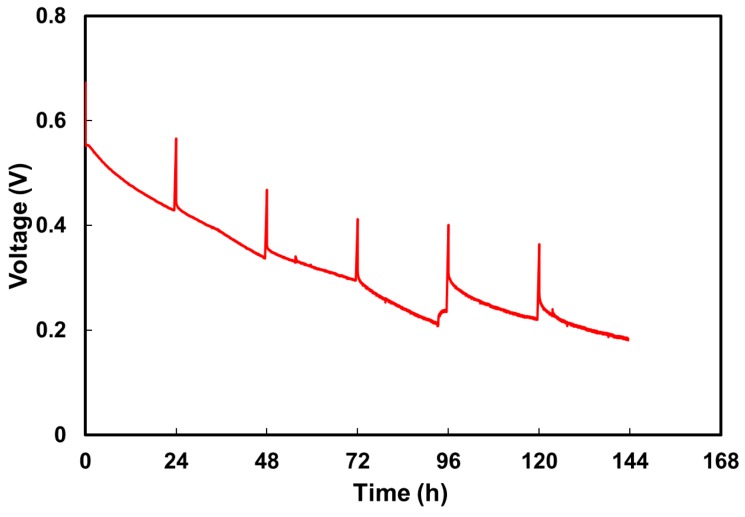
Long-term DMAFC voltage of PVA/40.5% ZIF-8 membrane at 60 °C at current density of 50 mA cm^−2^ using catalyst of 2 mg cm^−2^ Pt–Ru on carbon cloths and 1 mg cm^−2^ Pt on carbon cloths with micro-porous layer (MPL) for anode and cathode respectively (2 M methanol with 6 M KOH at a flow rate of 5 mL min^−1^ for anode fuel and humidified oxygen at a flow rate of 100 mL min^−1^ for cathode feed). The anode fuel was replenished after 93 h.

The PVA/40.5% ZIF-8 composite stability was employed for long-term cell testing at 60 °C. Figure 10 shows the change in the discharged voltage as a function of time with a constant current at 50 mA. The data were recorded over a continuous operating period of 144 h with 30 min off-periods every 24 h. During the first cycle, the cell potential decreased from 0.56 to 0.43 V, then the cell potential returned to 0.566 V during the off-period. In the fourth cycle, there was a larger voltage loss than in the previous three cycles. According to our previous work, Liao et al. [70] found a slight voltage loss resulting from the reduced fuel concentration. Thus, we changed the spent anode fuel into a fresh one and the cell potential increased from 0.208 to 0.238 V. Due to the voltage drop fluctuation shown in the fourth cycle, the discharge voltage curve was divided into two parts to calculate the individual decay rates. The decay rate was about 2.78 × 10^−3^ V h^−1^ in the first four cycles and 1.52 × 10^−3^ V h^−1^ in the last two cycles. The average decay rate was approximately 2.05 × 10^−3^ V h^−1^. By contrast, our prior study showed stable long-term DMAFC voltage results with the commercial E-tek gas diffusion electrodes (GDEs) with higher catalyst loads (5–6 mg cm^−2^) [26,46,70]. In addition, the produced CO_2_ and carbonate at the anode had little impact on the methanol/KOH solution pH value and the ionic conductivity [14,25]. The K_2_CO_3_ formation during 100 h of continuous operation in direct methanol alkaline fuel cells with recycling anode feed did not affect the cell performance. The amount of produced carbonate was negligible and the potassium salt was soluble in the aqueous anode feed [26]. This long-term electrode stability issue is currently under investigation. At the end of long-term cell testing, the fuel cell was dissembled and the membrane was examined. The PVA/ZIF-8 composite stayed intact during this long operating time, exhibiting stability in this task.

## 4. Conclusions

PVA/ZIF-8 composites were successfully prepared in this work from direct ZIF-8 suspension and PVA solution mixing to form high-loaded membranes without filler aggregation. A three-fold increase in ionic conductivity and 75% suppression in methanol permeability were found for the composite containing 40.5% ZIF-8 load. As the filler content was raised beyond 45.4%, adverse effects—reduced conductivity and increased methanol permeability—resulted, and the cell performance declined. A high peak power density of 173.2 mW cm^−2^ was achieved using the PVA/40.5% ZIF-8 at 60 °C with 1–2 mg cm^−2^ catalyst loading. The specific power density output is higher than that reported in other literature data.

## Figures and Tables

**Table 1:** Properties of the ZIF-8 nanoparticles and PVA/ZIF-8 composite.

Properties	PVA	PVA/25.40% ZIF-8	PVA/40.50% ZIF-8	PVA/45.40% ZIF-8
Polymer crystallinity (%)	38.09	34.18	31.87	31.33
KOH uptake (g g^−1^)	0.92	1.07	1.072	0.998
Conductivity ^1^ 30 °C (S cm^−1^)	0.0055	0.0158	0.0188	0.0147
60 °C (S cm^−1^)	0.0075	0.0174	0.0204	0.0156
Permeability ^2^ (10^−6^ cm^2^ s^−1^)	4.28	1.48	1.05	2.40
Selectivity ^3^ 30 °C	1292	10,533	17905	6125

^1^ Doped with 6 M KOH; ^2^ Methanol permeability from 2 M methanol as feed at 30 °C; ^3^ Ratio of ionic conductivity to methanol permeability.

**Table 2:** Peak power densities of DMAFCs using polymeric composite membranes at 50–60 °C.

Electrolyte Membrane	Anode Catalyst (Loading in mg cm^−2^)	Cathode Catalyst (Loading in mg cm^−2^)	Peak Power Density (mW cm^−2^)	Source
PVA	Pt/C (1)	Pt/C (1)	6	Fu et al. [27]
PVA/TiO_2_	Pt–Ru/C (4)	Pt/C (4)	8	Yang et al. [69]
PVA/fumed silica	Pt–Ru/C (5)	Pt/C (5)	39	Lue et al. [26]
PVA/multiwalled carbon nanotubes	Pt–Ru/C (5)	Pt/C (5)	39	Pan et al. [25]
PVA/graphene	Pt/C (5)	Pt/C (5)	46	Ye et al. [13]
PVA/carbon nanotubes (CNTs)	Pt–Ru/C (5)	Pt/C (5)	68	Lue et al. [68]
PVA/Fe_3_O_4_-CNTs	Pt–Ru/C (6)	Pt/C (5)	88	Lo et al. [50]
Tokuyama	Pt–Ru/C (8)	Pt/C (8)	55	Prakash et al. [73]
QPVA/Q-SiO_2_	Pt–Ru/C (4)	MnO_2_/C (4)	35	Yang et al. [72]
QPVA/chitosan	Pt–Ru/C (6)	Pt/C (5)	51	Li et al. [62]
Electrospun QPVA	Pt–Ru/C (6)	Pt/C (5)	54	Liao et al. [70]
CL-QPVA/GO-Fe_3_O_4_	Pt–Ru/C (6)	Pt/C (5)	55	Lin et al. [74]
Q-PVA/Q-chitosan	Pt–Ru/C (5)	Pt/C (5)	73	Liao et al. [46]
QPVA/fumed silica	Pt–Ru/C (5)	Pt/C (5)	88	Kumar et al. [71]
QPVA/GO-Fe_3_O_4_	Pt–Ru/C (6)	Pt/C (5)	200	Lin et al. [75]
PVA/40.5% ZIF-8	Pt–Ru/C (2)	Pt/C (1)	174	This work

QPVA: quaternized polyvinyl alcohol.
